# The role of the degenerate nucleotide binding site in type I ABC exporters

**DOI:** 10.1002/1873-3468.13997

**Published:** 2020-11-27

**Authors:** Thomas Stockner, Ralph Gradisch, Lutz Schmitt

**Affiliations:** ^1^ Institute of Pharmacology Center for Physiology and Pharmacology Medical University of Vienna Vienna Austria; ^2^ Institute of Biochemistry Heinrich Heine University Düsseldorf Germany

**Keywords:** ABC transporters, degenerate nucleotide binding site, transport cycle model, type I exporter

## Abstract

ATP‐binding cassette (ABC) transporters are fascinating molecular machines that are capable of transporting a large variety of chemically diverse compounds. The energy required for translocation is derived from binding and hydrolysis of ATP. All ABC transporters share a basic architecture and are composed of two transmembrane domains and two nucleotide binding domains (NBDs). The latter harbor all conserved sequence motifs that hallmark the ABC transporter superfamily. The NBDs form the nucleotide binding sites (NBSs) in their interface. Transporters with two active NBSs are called canonical transporters, while ABC exporters from eukaryotic organisms, including humans, frequently have a degenerate NBS1 containing noncanonical residues that strongly impair ATP hydrolysis. Here, we summarize current knowledge on degenerate ABC transporters. By integrating structural information with biophysical and biochemical evidence of asymmetric function, we develop a model for the transport cycle of degenerate ABC transporters. We will elaborate on the unclear functional advantages of a degenerate NBS.

## Graphical abstract

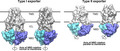

## Abbreviations

**NBD**, nucleotide binding domain

**NBS**, nucleotide binding site

**TMD**, transmembrane domain

Research into ATP‐binding cassette (ABC) transporters has started with the initial discovery of cancer cells showing resistance to colchicine treatment, and it was early on discovered that the protein responsible for the resistance must be an ATP‐driven transporter termed permeation glycoprotein (P‐glycoprotein), later systematically named ABCB1 [1, 2]. Sequence analyses have identified ABC transporters as constituting one of the largest protein families that ranges from Archaea to humans. While in bacterial genomes ABC transporters include exporters and importers, ABC transporters are typically exporters in eukaryotic organisms, with known exceptions being the human ABCA4 [3, 4], human ABCD4 [5], and a few plant ABC transporters [6, 7, 8, 9]. The first structure of an ABC transporter domain was the crystal structure of the nucleotide binding domain (NBD) of the histidine importer HisP [10], while the structure of the vitamin B12 importer BtuCD [11] was the first structure of a complete ABC transporter. The structure of Sav1866 [12] revealed the fold of type I ABC exporters. These transporters have been solved in three main conformations: (a) in the inward‐facing state, which is characterized by partially or completely separated NBDs [13, 14], while the substrate‐binding site in the transmembrane domains (TMDs) is accessible from the cytosol or the inner membrane leaflet, but sealed toward the extracellular site; (b) in an occluded state, which shows a nucleotide‐stabilized NBD dimer and sealed access path(s) to the substrate‐binding site [15, 16]; and (c) in the outward‐facing state, which also shows a nucleotide‐stabilized NBD dimer, while the substrate‐binding site opens toward the extracellular site, therefore allowing for substrate release. These structures confirmed earlier sequence analysis results and biochemical data showing that the minimal functional unit of an ABC transporter consists of two TMDs and two NBDs. The two TMDs associate to form the substrate recognition site and the path for substrate translocation. The two NBDs energize substrate transport by binding and hydrolyzing two ATP molecules. These four domains can be encoded by a single gene (full‐size transporter), an assembly from two half transporters (half‐size transporter), each consisting of a TMD and an NBD, or the four domains can be independent protein chains that associate to form a functional transporter. ABC transporters can transport an astonishing variety of substrates ranging from small ions such as chloride or manganese to nutrients such as amino acid, sugars, or vitamins to large proteins such as Microcin J25 [17], HlyA [18], or LapA with a molecular weight of 900 kDa [19].

ATP hydrolysis is carried out by composite ATP hydrolysis sites or nucleotide binding sites (NBSs) that are symmetrically located in the interface between both NBDs and formed by residues from both NBDs. This interface harbors the conserved sequence motifs of this superfamily, which are all involved in binding and/or hydrolysis of the bound nucleotide triphosphate. On a molecular level, one NBS is formed by the Walker A and B motif, the A‐, Q‐ and H‐loop of one NBD and the signature sequence, and the X‐loop of the second NBD. ATP acts as a molecular glue that brings both NBDs together and holds them in close association, while the D‐loops of both NBDs stabilize the dimer by multiple interactions in the center of the NBD dimer interface. The glutamate residue in the Walker B motif was shown to be essential for ATP hydrolysis, and consequently, its mutation to glutamine leads to functionally impaired transporter variants [20]. Without hydrolysis, the ATP‐bound conformations are very stable, locking the ABC transporter in the ATP‐bound state. The majority of ABC transporters have two functional ATP‐binding sites that are capable of hydrolyzing ATP. If the conserved motifs of one of these two ATP‐binding sites contain noncatalytically active amino acids, this NBS would no longer be able to hydrolyze ATP to an extent that would sustain physiologically relevant transport [21, 22]. Consequently, such a NBS would be called ‘degenerate’. However, the consequences of one impaired site are still unclear with respect to mechanistic consequences and implications for the transport cycle. These transporters are active and must therefore have established a new function or mechanism, in which the degenerate NBS sustains active substrate translocation across a biological membrane. Some of the proposed transport cycle models that have been derived for ABC transporters containing two hydrolysis competent NBSs are not compatible with an ABC transporter harboring a degenerate site, because these models (a) require the hydrolysis of two ATPs, (b) an obligate alternation of ATP hydrolysis between NBS1 and NBS2, or (c) impose complete NBD separation (for more details see below). An indication in support of an acquired new function is sequence conservation, because sequences of the degenerate NBS have not deviated in ABC transporter orthologs, showing that evolutionary pressure remained high in the degenerate site. The newly acquired function remains unclear, but indirect evidence suggests that ATP might serve as a scaffold acting like a permanent glue in the degenerate site, keeping the NBS in a closed conformation over several iterations through the transport cycles [21, 22, 23, 24, 25].

## The nucleotide binding domains

**Fig. 1:**
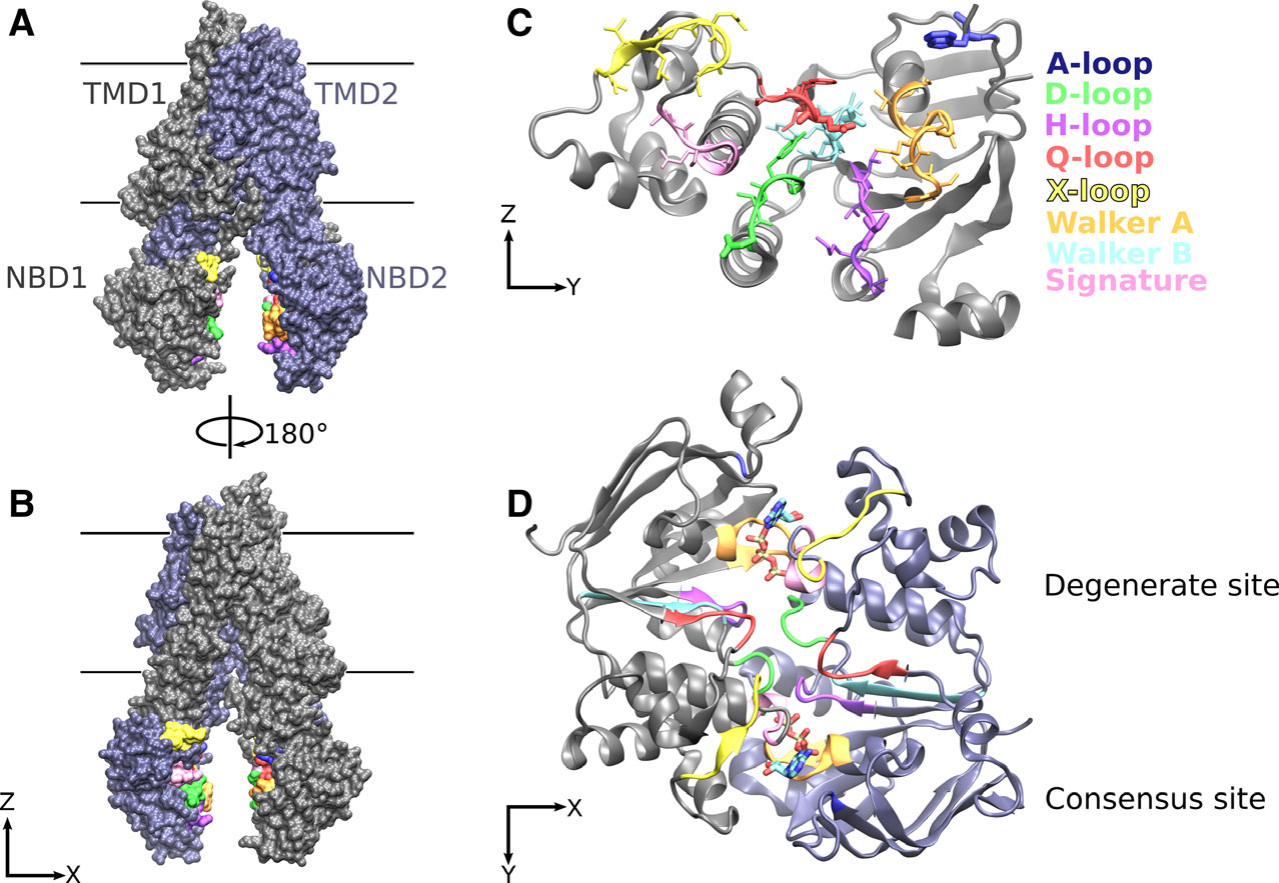
ABC transporter structure. (A, B) Side views of inward‐facing ABCC7 (PDB ID: 5UAK [70]) showing the TMD1 and NBD1 in gray, while TMD2 and NBD2 are in violet. The NBD motifs are colored as labeled in panel C. (C) The NBD motifs are highlighted by color and their side chains shown as sticks. The NBD is oriented to show the NBD surface that constitutes the NBD‐NBD interface. (D) Nucleotide‐bound ABCC7 (PDB ID: 6MSM [68]) structure is used to visualize the closed NBD dimer. The NBDs are seen from the TMD domain. For simplicity, the TMD is not shown. All noncanonical motifs in the NBD1 and in NBD2 are clustered at the degenerate NBS1.

The NBDs of ABC transporters consist of two subdomains: a larger Rec‐like domain and a smaller helical domain (Fig. 1). Initial structures of isolated NBDs revealed its fold [10, 26, 27, 28, 29, 30], while structures with nucleotides bound to the NBD dimer highlighted the NBSs that are formed in the interface between the two NBDs [31, 32] (Fig. 1). The structure of the NBD dimer revealed that the conserved motifs are all located at the NBD dimer interface. The glutamate in the Walker B motif [33] has been proposed to directly interact with the γ‐phosphate of ATP, the magnesium ions, and/or to position an activated water molecule for a nucleophilic attack on the phosphate bonds of ATP. The Q‐loop [34] includes a conserved glutamine that also interacts with and senses the presence of the γ‐phosphate of ATP. The Q‐loop is also engaged with the intracellular loop of the TMDs and has therefore been proposed to play a role in the transmission of signals between the TMDs and the NBDs. The H‐loop [35, 36] is proposed to interact with the γ‐phosphate and has been recognized for playing an important role in ATP hydrolysis. The signature sequence [37] interacts with the γ‐phosphate of ATP and forms hydrogen bonds to the ribose ring of ATP. The Walker A [33] motif interacts with the phosphates of ATP and provides the key binding sites for the α‐ and ß‐phosphates. The A‐loop [38] typically contains an aromatic residue that forms π stacking interactions with the adenine base to stabilize the bound nucleotide. The residues immediately following the X‐loop [12] stabilize the adenine base from the other site. The D‐loop [37, 39] is located in the center of the NBD‐NBD interface and makes extensive interaction with its counterpart on the other NBD. It was shown that the D‐loop is important for NBD‐NBD interactions and for ATP hydrolysis, probably by providing a pathway for communication between the two NBS and by properly orienting the two NBDs relative to each other.

### Sequence comparison

The human genome encodes 48 ABC genes: Four of them are proteins involved in ribosome function and regulation (ABCE1‐3 and ABCF1), while 44 encode transmembrane proteins. These assemble into 42 membrane proteins, because four of these genes encode half‐size transporters that associate to form heterodimeric transporters (ABCB2/B3 and ABCG5/G8). Thirty‐four of the membrane proteins are transporters, while one is an ATP‐gated chloride ion channel (ABCC7) and two (ABCC8 and ABCC9) are sulphonylurea receptors that regulate the potassium ion channels Kir6.1 and Kir6.2.

**Fig. 2:**
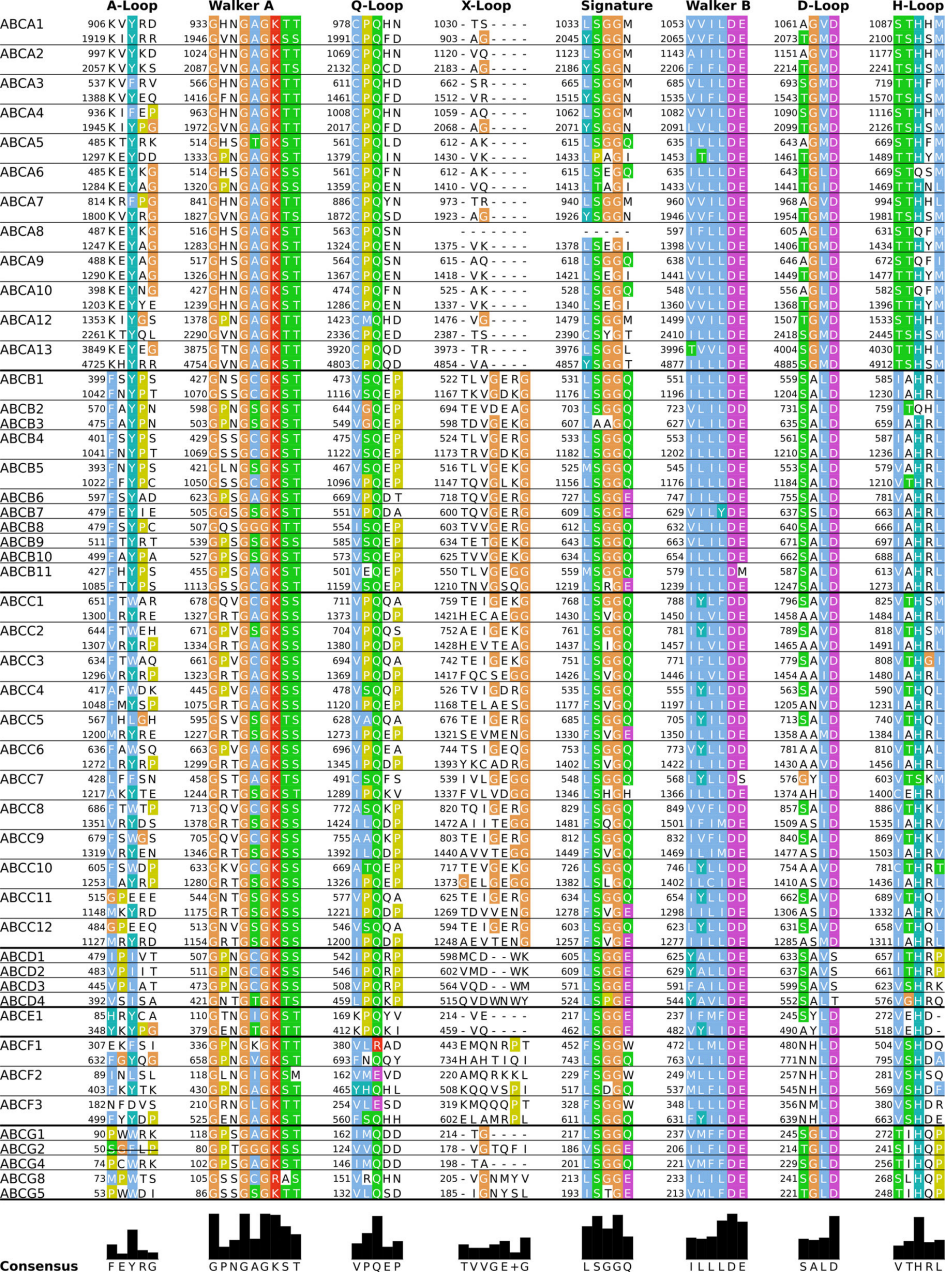
Sequence alignment of the NBD motifs of all human ABC proteins. Canonical isoforms of each nonhomodimeric transporter were aligned, and NBD1 and NBD2 shown above each other. The numbers indicate the sequence position, and the bar graph at the bottom indicates sequence conservation.

Sequence comparisons of ABC transporters show that the TMDs have heavily diverged during evolution, which is most likely associated with the broad spectrum of transported substrates that ranges in size from a single ion to an astonishing 1.5 MDa [40]. In contrast, the NBDs (Fig. 2) that bind and hydrolyze ATP remained similar to each other with respect to sequence and consequently to three‐dimensional fold. Interestingly, while the NBDs are the most conserved region of the transporter, 21 of the 29 full‐length or heterodimeric ABC proteins present in the human genome harbor a degenerate NBS. The deviations from the consensus sequence are distributed over both NBDs, because each NBS is a composite arrangement of conserved sequence motifs (Fig. 1D). The degenerate NBS is always NBS1 in the full‐length human ABC proteins. Deviations from the consensus sequence are present in all members of the ABCC subfamily and in a subset of the ABCA, ABCB, and ABCG subfamilies. Only the ABCD subfamily of human ABC transporters does not include transporter with a degenerate NBS1. Heterodimeric ABC transporters could in principle be aligned in two ways with their full‐length counterpart. To compare full‐length and heterodimeric transporters with a degenerate NBS, the transporters are structurally and functionally aligned by overlaying their degenerate NBS with NBS1 of full‐length transporters. A comparison of human ABC transporter sequences shows a nonrandom pattern and a correlation between the deviations from the consensus sequence and the type ABC transporter subfamily. The functional origin of this pattern is still enigmatic, but it is tempting to speculate that it might be associated with the type of motions associated with the transport cycle.

Mutations of the signature sequence in NBD2 are present in all degenerate NBSs of the human genome. The second most frequently observed change, which results in a degenerate NBS, is the exchange of the C‐terminal glutamate residue of the Walker B motif to an aspartate. This is a very conservative mutation, because it replaces the negatively charged glutamate with the negatively charged aspartate, which only differs by reduction of the length of the amino acid side chain by one methylene moiety. The NBSs carrying such a mutation seem still able to hydrolyze ATP [41, 42], but the rate of hydrolysis is much slower than the overall transport cycle, indicating that ATP hydrolysis in NBS1 cannot directly sustain transport. One exception to the typical conservative glutamate to aspartate deviation (as present in most ABCB and ABCC family members) is NBS1 of the bile salt export pump (BSEP or ABCB11), where the glutamate of the Walker B motif is replaced by a methionine. ATP hydrolysis seems to be completely blocked in the presence of methionine [43]. The second exception from the rule is ABCC7, where the catalytic glutamate is replaced by a serine. A mutation of the glutamate in the Walker B is frequently associated with deviations in the H‐loop [30] as present in several transporters from the ABCA subfamily, in the ABCB2/B3 heterodimer, while in the ABCC subfamily only in ABCC7. The degenerate NBS of the human ABCG5/G8 heterodimer differs in the pattern, as changes in the signature sequence are accompanied by changes in the Walker A motif, replacing the lysine residue in the Walker A with an arginine.

## Conformational (a)symmetry in crystal and cryo‐EM structures

ATP hydrolysis is asymmetric in ABC transporters with a degenerate site, leading to a high energy state [44, 45] in the canonical NBS2 (MgADP + Pi) after hydrolysis of ATP, while degenerate NBS1 contains the nonhydrolyzed ATP. Accordingly, NBD separation will most likely be asymmetric, as MgATP bound to NBS1 represents a low energy state [44, 45] that stabilizes the closed NBS1 and thus glues the NBDs together. The motions of NBD separation could in theory still be coordinated and symmetric, if other parts of the transporter would enforce such symmetric motion, similar to hemoglobin, which seems to operate using concerted motion of all four hemoglobin subunits in response to oxygen binding [46].

**Fig. 3:**
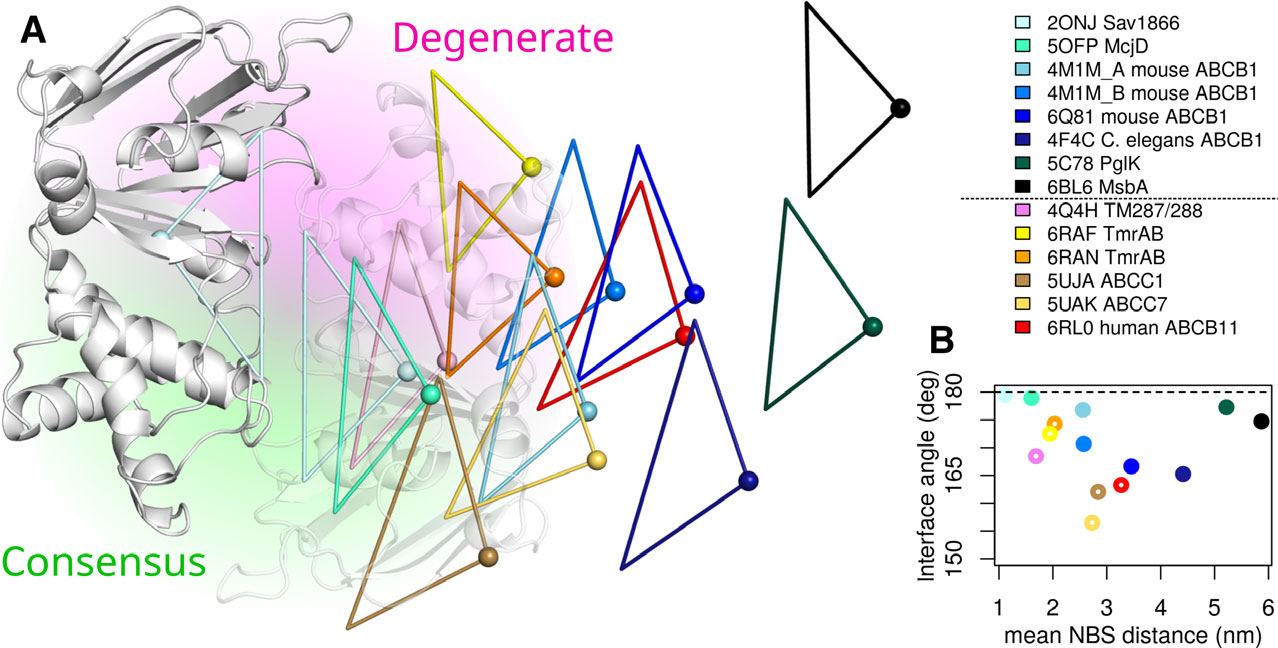
Relative orientation of NDBs. (A) Representative inward‐facing conformations of type I ABC transporter have been structurally aligned at NBD1, shown in gray. The second NBD is shown in white only for Sav1866. Each NBD is reduced to a simplified representation of a triangle that encodes the domain orientation. The corners of the triangle are the center of mass of the NBD, indicated by a sphere, the first residue in the signature sequence helix and the last residue in the helix of the Walker A motif. Transporters harboring a degenerate NBS have warm colors, while transporters with two canonical NBSs have cold colors. (B) Quantification of relative orientation and NBD distances, using the same color code as in A. Open circles mark transporters with a degenerate side.

The currently available structures of ABC exporters (Table 1) with a type I fold containing a degenerate NBS1, including TM287/288, TmrAB, ABCB2/B3, ABCB11, ABCC1, ABCC7, ABCC9, and ABCC10, suggest that asymmetric motions are encoded in the primary and the tertiary structure (Fig. 3). This notion is supported by structures in the absence of nucleotides and thus without their driving forces, showing NBDs at various degrees of separation and, importantly, asymmetric separation of the NBDs. Equally important, NBD separation is clearly more asymmetric in ABC transporters that have a degenerate NBS (Fig. 3). ABCB11 and both ABC proteins from the ABCC subfamily (ABCC1 and ABCC7) show rotations of up to 20°. In addition to relative rotations, ABCC1 and TmrAB show large lateral motions that misalign the NBD motifs of the NBSs.

**Table 1:** Degenerate ABC transporters for which structural information is available in the protein data bank (www.rcsb.org). See text for further details.

ABC transporter	PDB entry	Organism	Method	Environment	Conformation	Citation
ABCB2/B3	5U1D	Human	cryo‐EM	Detergent‐solubilized	Inward‐facing	[62]
ABCB11	6LR0	Human	cryo‐EM	Detergent‐solubilized	Inward‐facing	[63]
ABCC1	5UJA, 5UJ9	Bovine	cryo‐EM	Detergent‐solubilized	Inward‐facing	[65]
	6BHU	Bovine	cryo‐EM	Detergent‐solubilized	Outward‐facing	[66]
	6UY0	Bovine	cryo‐EM	Detergent‐solubilized	Outward‐facing	[67]
ABCC7	5UAK	Human	cryo‐EM	Detergent‐solubilized	Inward‐facing	[70]
	5W81	Zebra‐fish	cryo‐EM	Detergent‐solubilized	Occluded	[69]
	6D3R, 6D3S	Chicken	cryo‐EM	Detergent‐solubilized	Inward‐facing	[72]
	6O1V, 6O2P	Human	cryo‐EM	Detergent‐solubilized	Occluded	[155]
	6MSM	Human	cryo‐EM	Detergent‐solubilized	Occluded	[68]
	5UAR	Zebra‐fish	cryo‐EM	Detergent‐solubilized	Inward‐facing	[71]
TM287/288	6QV0, 6QV1, 6QV2, 6QUZ	*Thermotoga maritima*	X‐ray	Detergent‐solubilized	Outward‐facing	[47]
	3QF4	*Thermotoga maritima*	X‐ray	Detergent‐solubilized	Inward‐facing	[13]
	4Q4H, 4Q4J, 4Q4A	*Thermotoga maritima*	X‐ray	Detergent‐solubilized	Inward‐facing	[48]
TmrAB	5MKK	*Thermus thermophilus*	X‐ray	Detergent‐solubilized	Inward‐facing	[50]
	6RAF, 6RAN, 6RAM, 6RAI, 6RAG, 6RAJ, 6RAI, 6RAL, 6RAK	*Thermus thermophilus*	cryo‐EM	Detergent‐solubilized	Entire transport cycle	[52]
ABCC8	5YWA, 5YKF, 5YKG, 5YW7, 5YD8, 5YW9	Hamster	cryo‐EM	Detergent‐solubilized	Inward‐facing	[156]
	5YDB, 5YWC, 5YWD, 5YW9	Hamster	cryo‐EM	Detergent‐solubilized	Occluded	[156]
	6BAA	Hamster	cryo‐EM	Detergent‐solubilized	Inward‐facing	[157]
	6JB1	Hamster	cryo‐EM	Detergent‐solubilized	Inward‐facing	[158]
	6PZA, 6PZB, 6PZC, 6PZI, 6PZ9	Hamster	cryo‐EM	Detergent‐solubilized	Inward‐facing	[159]
	6C3O, 6C3P	Human	cryo‐EM	Detergent‐solubilized	Occluded	[160]
ABCG5/G8	5DO7	Human	X‐ray	Detergent‐solubilized	Inward‐facing	[145]

**Fig. 4:**
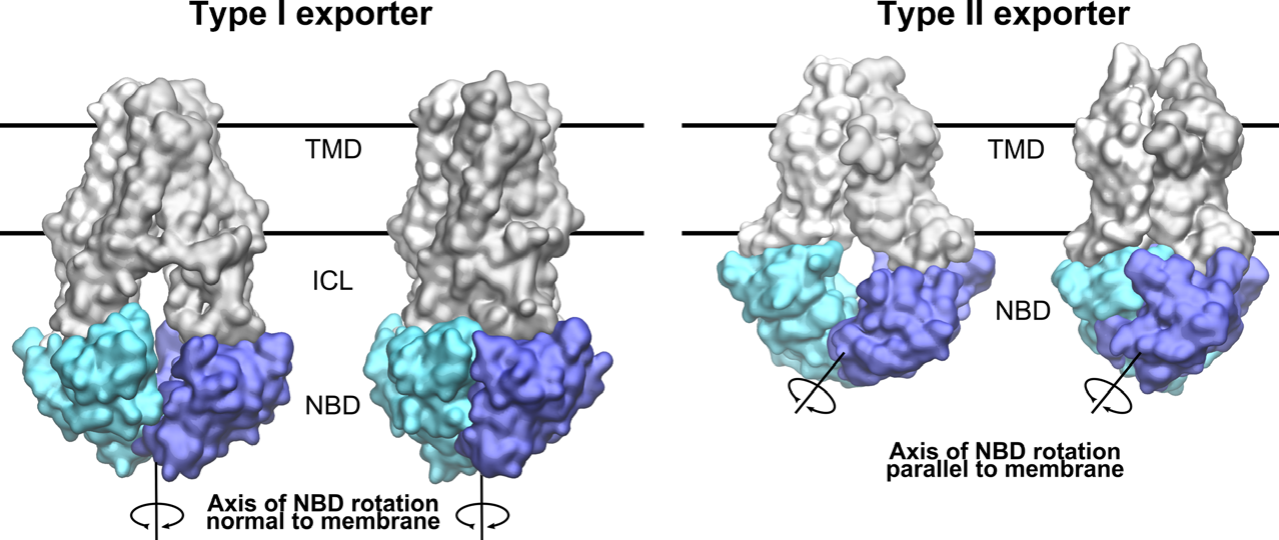
Axis of NBD rotation. The main axis of NBD rotation as a consequence of ATP binding and hydrolysis that is associated with the conformational changes in the TMDs is oriented normal to the membrane in type I exporter and parallel to the membrane in type II exporters.

In contrast to type I exporters, the heterodimeric type II exporter ABCG5/G8 shows symmetric NBD separation similar to ABCG2, despite the degeneration of NBS1. Structures of transporter from the ABCA (ABCA1) and ABCG (ABCG2 and ABCG5/G8) subfamilies show additional extensive interaction surfaces of their NBDs at the C‐terminal ends, which clamp the NBDs together, similar to ABC importers. A mechanical consequence is a relocation of the main axis of NBD rotation that runs parallel to the membrane in ABCA and ABCG transporters, but perpendicular in ABCB and ABCC and most likely ABCD transporters (Fig. 4). Additional structural and spectroscopic experiments will be needed to confirm symmetric motions for ABCG5/G8.

### TM278/288

TM287/288 was the first crystallized heteromeric ABC exporter that contained a degenerate site. In the presence of ATP‐γS, TM287/288 adopted an outward‐facing conformation [47], which is very similar to the conformation observed for Sav1866 [12], indicating that transporters with a degenerate NBD can reach the same ATP‐bound outward‐facing state as transporters with two canonical NBSs. The inward‐facing state of TM287/288 was also the first type I exporter structure that showed NBD‐NBD contacts and unmasked an asymmetric conformation of the NBD dimer as well as of the TMD dimer [13]. Asymmetry was subsequently confirmed by electron paramagnetic resonance (EPR) measurements [48, 49], showing that the degenerate site samples closed‐like conformations also in the apo state and in the absence of Mg^2+^. In contrast, the consensus NBS2 requires the presence of MgATP to reach the closed state. Interestingly, an inward‐facing structure showed only one molecule of AMP‐PNP bound to the degenerate NBS1, while being very similar to the apo conformation. An exception is thereby the D‐loop of TM288, which reaches over and interacts with AMP‐PNP bound to NBS1, markedly deviating from the typical D‐loop conformation. Typically, the D‐loop shows no direct interaction with the nucleotide in the occluded state. It will remain to be seen if this feature of the D‐loop is TM287/288 specific or generally relevant for sensing the presence of ATP in the inward‐facing transporter and promoting NBD closure. This interpretation is supported by alanine mutations of the interacting aspartate of the D‐loop of TM288, which showed reduced ATP affinity and lower ATP hydrolysis activity [48].

### TmrAB

Similar to TM287/288, the inward‐facing structures of heterodimeric TmrAB [50, 51] show an asymmetric TMD and NBD dimer arrangement, while the NBDs are in a pseudoseparated conformation. The NBD dimer of TmrAB carries an additional large leucine zipper‐like motif at the C terminus that stabilizes the NBD dimer also when both NBSs are fully separated. Asymmetry and lateral displacement of the NBD dimer in TmrAB is larger than in TM287/288, leading to a misalignment of the NBD motifs from the arrangement necessary for ATP binding and hydrolysis. A recent series of eight cryo‐EM structures [52] captures nucleotide‐bound TmrAB in various conformations. These structures are nucleotide bound, except for the asymmetric inward‐open structures. The ATP‐bound prehydrolytic and the vanadate trapped posthydrolytic state were both determined in an outward‐occluded and outward‐open conformation. Unexpectedly, the two outward‐occluded and the two outward‐open structures, while differing in their nucleotide binding state, were structurally identical within experimental resolution. It is tempting to infer conformational motions between the misaligned apo inward‐facing conformation for reaching a canonical ATP‐bound NBD dimer conformation that brings all NBSs motifs into close proximity [50], but it remains to be proven that the observed conformations of laterally displaced NBDs are part of the transport cycle. The high turnover rate of TmrAB would indicate smaller amplitude motions, while the necessary disruption of the leucine zipper‐like helix arrangement will require large energetic input for fast conformational changes. EPR measurements [53] show similar to TM287/288 that NBD motions of TmrAB are asymmetric. The degenerate NBS is more likely to reach the close state, which contrasts the structural finding that would indicate a more likely opening of the degenerate site. Similar to TM287/288, EPR data show that nucleotide dependent NBS closure is coupled to motions of the TMDs to reach the outward‐facing facing state.

### Multidrug resistance transporter 1 (MDR1, P‐glycoprotein or ABCB1)

Structures for four of the five nonsymmetric human transporters from the ABCB subfamily have been solved (ABCB1, ABCB2/B3, ABCB4, ABCB11). Human ABCB1 [16, 54, 55] and homologues (mouse [56, 57] *Caenorhabditis elegans* [58] *C. merolae* [59, 60]) are full‐length transporters, in which the sequences of the N and the C‐halves have diverged from an initial putative gene duplication, maintaining pseudo twofold symmetry, but the full‐length transporter can also be interpreted as a heterodimer with two canonical NBSs. The conformations of the outward‐facing and the occluded state are largely symmetric showing pseudo twofold symmetry, while Fig. 2 suggests that the inward‐facing structures of ABCB1 and its homologues show deviations from symmetry, as the relative NBD‐NBD arrangement and also the direction of NBD‐NBD separation differ. Interestingly, the structure of mouse ABCB1 showed two different conformations in the same crystal. Together, these ABCB1 structures suggest that the inward‐facing structure might show asymmetric NBD movements despite having two canonical NBSs. It will remain to be seen if all observed conformations of ABCB1 are part of the transport cycle or if a subset of these structures are stabilized in nonphysiological relevant conformations.

### Transport associated with antigen processing (TAP or ABCB2/B3)

The structure of the transporter associated with antigen processing (TAP or ABCB2/B3) has been solved by cryo‐EM at medium resolution (overall 7.2 Å) [61] as part of the herpes virus inhibitor ICP47 stabilized peptide loading complex. This complex is essential for adaptive immunity because loading antigen peptides onto the major histocompatibility complex I. The medium resolution structure of ABCB2/B3 is visible in the cryo‐EM as a nanodisc inserted heterodimer. The cryo‐EM density suggests that the NBDs are not fully associated, because their interaction surface is minimal. The isolated structure of ABCB2/B3 was solved in complex with an inhibitory peptide from the herpes simplex virus [62] that stabilized ABCB2/B3 in a conformation with widely separated NBDs, but the passage for peptides to the ER lumen is not completely sealed. Both structures of ABCB2/B3 are stabilized by the viral inhibitory peptide ICP47, but the conformations of ABCB2/B3 differ between the isolated transporter and in complex with the peptide loading complex. The overall structural difference shows that the NBDs of ABCB2/B3 separate in the absence of the nanodisc and/or the peptides loading complex. It remains to be seen if this environment dependency is unique to ABCB2/B3 or if shared with other type I exporters.

### Bile Salt Export Pump (BSEP or ABCB11)

The ATPase activity of detergent‐purified ABCB11 can be stimulated by substrates such as glycocholic acid, taurocholic acid, or taurodesoxycholic acid 6‐ to 7‐fold while known inhibitors such as rifampin or glibenclamide also stimulate the ATPase activity [63]. The cryo‐EM structure of ABCB11 [63] shows an asymmetric inward‐facing conformation in which the degenerate NBS1 has a larger separation than the canonical NBS2. The inward‐facing conformation is stabilized by the N‐terminal first 30–45 residues, which adopt a helix‐turn‐helix motif that is inserted between the TMDs and serves as a conformation‐stabilizing wedge. This conformation is most likely nonphysiological because its truncation did not change the ATPase activity, while a large influence on the ATPase activity would be predicted from the structure as NBD‐NBD association is prevented, while a prerequisite to form the hydrolysis competent NBD dimer.

### Multidrug resistance‐associated protein 1 (MRP1 or ABCC1)

The multidrug resistance protein 1 (MRP1 or ABCC1), which harbors a degenerate NBS similar to all other transporters from the ABCC subfamily, is a promiscuous transporter that recognizes a diverse range of substrates [64]. The structure of the bovine ABCC1 has been determined in multiple conformations: In a wide‐open inward‐facing conformation in the apo state [65], a slightly less wide‐open inward‐facing state in the presence of a leukotriene C_4_ ligand [65], while the third conformation [66], the outward‐facing state, was obtained in the presence of ATP after mutation of the Walker B glutamate to glutamine. Both inward‐facing conformations show a strong relative displacement of the NBDs that leads to a misalignment between the Walker A motif and the signature sequence. In addition to NBD displacement, the structures also show a large rotation of NBD1 relative to NBD2 that results in a larger opening of the degenerate NBS1. The outward‐facing structure resembles the conformations observed for other ABC transporters with two canonical NBSs. Also, the structure of wild‐type bovine ABCC1 obtained in the presence of ATP by fast quench freezing showed basically identical geometries for NBS1 and NBS2, despite ATP being hydrolyzed to ADP in NBS2.

Single molecule experiments report on distances between labels attached to the transporter. The distances observed for detergent‐purified wild‐type bovine ABCC1 [67] were compatible with the conformations of the cryo‐EM structures, but structural asymmetry was not investigated. The NBDs are far apart in the absence of nucleotide, the distance decreases by the addition of the leukotriene C_4_ ligand, while only the presence of ATP induces a conformation in which the NBD is in close contact. Titration and kinetic experiments showed that the addition of ATP leads to an outward‐facing state at concentrations below the *K*_D_ of ATP, but only in a subset of tested ABCC1 transporters. Increasing concentration of ATP accelerates the transition to the outward‐facing state, while slowing down the return from the outward to the inward‐facing state. Unexpectedly, no difference for returning to the inward‐facing state was observed between wild‐type ABCC1 and the Walker B glutamate ABCC1.

While providing very valuable information on protein structure and dynamics, the use of detergent‐purified transporters seems to have changed transporter properties relative to the situation in cellular membranes. *K*_M_ values, substrate binding, and turnover numbers change [65]. Earlier biochemical data [22] have shown that ABCC1, ABCC7 [21, 23, 24], and ABCC9 [25] bind ATP at both NBSs, while kinetic analysis revealed that ATP remains tightly bound to the degenerate NBS1 over timescales of minutes. Such a slow dissociation rate indicates that the degenerate NBS1 does rarely dissociate and ATP remains bound, while cycling 20–30 times through the transport cycle. This discrepancy also suggests that high‐resolution data need to be carefully scrutinized, if the environment of the measurements differs from its native membrane environment. These biochemical data question the physiological relevance of the wide‐open, inward‐facing state as observed for several ABC transporters in the absence of ATP. From kinetic considerations, it would be inefficient for the transporter to remain mostly in the outward‐facing state in the presence of typical cytosolic ATP concentrations, while rarely reaching an inward‐facing state that can accept substrates, because thereby a strong reduction in the effective concentration of substrate‐accessible transporters would occur.

### The cystic fibrosis transmembrane conductance regulator (CFTR or ABCC7)

The cystic fibrosis transmembrane conductance regulator (CFTR or ABCC7) is an ATP‐gated nonselective anion channel, which is a function unique among ABC proteins. ABCC7 carries a glutamate to serine mutation in the Walker B motif, while all other proteins of the ABCC subfamily harbor a glutamate to aspartate mutation in NBS1. ABCC7 differs from all other ABC proteins by having a regulatory domain inserted in the linker connecting NBD1 with TMD2, which controls channel gating through phosphorylation status. The NBDs of the ATP‐bound outward‐facing conformation of the Walker B glutamate to glutamine mutant of human ABCC7 [68] show indistinguishable ATP binding to NBS1 and NBS2, in a conformation that is very similar to ABCC1. The cryo‐EM structure of the ATP‐bound mutant ABCC7 from danio rerio shows a slight asymmetry, because of a small opening (~ 0.2 nm) of NBS1 [69].

The inward‐facing structures of human and danio rerio ABCC7 [70, 71] are also in the ATP‐bound state and show a NBD‐NBD distance that increased by ~ 2 nm as well as a pronounced rotation of the NBDs relative to each other. In contrast, the inward‐facing structure of chicken ABCC7 [72] shows a smaller separation, almost no relative rotation of the NBD, but a large misalignment of the NBD motifs. Interestingly, the detergent‐purified human and danio rerio ABCC7 showed a membrane‐exposed arginine residue in TMH8 that locates the positively charged guanidinium side chain into the center of the membrane in the cryo‐EM structures. Translocation of an arginine side chain from water to the center of the membrane was shown to be energetically very unfavorable, requiring up to ~ 60 kJ·mol^−1^ [73].

The slight separation of NBS1 in the ATP‐bound danio rerio structure is intriguing, because similar to ABCC1, biochemical data on ABCC7 show that ATP remains bound to NBS1 for several minutes while the ABCC7 cycles through several channel gaining events, suggesting that NBS1 does not open under physiological conditions [21, 23, 24].

In summary, sequence and structural data support the notion that asymmetry of the NBSs and consequently of ATP hydrolysis and energy provision has created evolutionary pressure. This has led to a divergence in the mechanics and thus in the motions to take advantage of asymmetric NBSs function. It has not yet been possible to identify systematic sequence changes in the TMDs that are associated with the response to the degeneration of NBS1. The asymmetry seen in apo inward‐facing full transporters shows that the inward‐facing state, associated with substrate binding, is asymmetric, while these transporters convert to a largely symmetric conformation in the outward‐facing state. Because of such complex conformational behavior, integration of sequence information, structure determination, biochemical and biophysical measurements, and dynamic data will be required to decipher the still largely hidden mechanistic features of asymmetric ABC transporter function.

## Biochemical evidence for asymmetry

Sequence and structural analysis of ABC transporters showed that the deviation from the consensus sequence maps to NBS1. Modification of the catalytic glutamate of the Walker B sequence in the canonical NBS1 blocks transport function. In contrast, NBS1 of degenerate transporters is much more tolerant to mutations, as modifications often show only minor changes in function.

### ABCB1

Experimental data of wild‐type ABCB1 revealed that interactions of ATP with ABCB1 can be asymmetric, but also showed that NBS1 and NBS2 are functionally equivalent. Obviously, ABCB1 is not a degenerate transporter, but the wealth of data is important for the following discussion. The inferences are derived from data demonstrating that ABC transporters bind two molecules of ATP to the symmetry‐related NBSs [74], while a mutation of either Walker A motif [75, 76, 77] or the catalytic glutamate of the Walker B motif [20] block transporter function. Only one nucleotide bound to either NBS could be detected using the ATP analog ATP‐γS [78], vanadate trapping [79, 80], or binding of MgADP + BeF_4_ [80]. UV irradiation detected binding of 8‐azido‐ATP to both NBSs [79], while photochemical cleavage of the vanadate trapped state showed random cleavage of either NBS, but not of both NBS simultaneously [81].

Mutations of the Walker B glutamates had different outcomes: While mutation of both Walker B motif glutamates to glutamines, aspartates, or alanines led to the same residual ATPase activity, these ABCB1 variants differed in their capability to retain bound ATP [42], in the *K*_D_ of ATP binding, in verapamil stimulated ATPase activity, and in vanadate trapping. The single Walker B glutamate mutants revealed that NBS1 or NBS2 is not completely identical [41]: The verapamil stimulated ATP hydrolysis of single glutamate to glutamine mutants in NBS1 or NBS2 differed 2‐ to 3‐fold, the *K*_m(MgATP)_ differed 5‐fold, while ADP is more likely retained in the Walker B mutant of NBS1 compared to NBS2. Insertion of methionine (as present in ABCB11) in place of the Walker B glutamate of NBS1 abolished ATPase activity and substrate transport [82]. Inserting all four residues by which NBS1 of ABCB11 differs from NBS1 of ABCB1 resulted in an ABCB1 variant that can hydrolyze ATP and is substrate transport competent [82], showing that ABCB1 can in principle be active with a degenerate NBS1.

Taken together, these data indicate that ABCB1 binds two molecules of ATP, but states associated with ATP hydrolysis can be detected in only one of the NBSs, suggesting that the state associated with ATP hydrolysis is asymmetric. Interestingly, asymmetry was also reported for the homodimeric transporters MsbA [83] and BmrA [84]. In addition to random asymmetric occlusion, cooperativity was detected between the two NBSs [31, 85, 86, 87, 88, 89], supporting the notion of functional asymmetry without any detectable preference of one NBS over the other.

### ABCB2/B3

The NBDs of ABCB2/B3 have been investigated in isolation. Rat NBD1 forms dimers in a MgATP‐dependent manner, while remaining monomeric in the presence of MgADP [29]. In contrast, the human NBD1 does not dimerize in the presence of MgATP [29, 90]. Mutational analysis showed that the difference is confined to the D‐loop, where rat and human sequences differ by an aspartate to asparagine change [29]. After replacing the aspartate of the human sequence with the asparagine as present in the rat sequence, the human ABCB2 NBD also showed MgATP‐dependent dimerization, highlighting the importance of the D‐loop for NBD dimerization. Full‐length human ABCB2/B3 transporter remained active after introducing mutations to the degenerate NBS, while mutations to the consensus NBS were not tolerated [91, 92, 93], suggesting that the degenerate NBS differs in function from the canonical NBS. Inserting the consensus residues into the degenerate NBS1 reduced ATP hydrolysis and transport function, while for isolated isolated NBD of ABCB2, these mutations increased the ATP hydrolysis rate [90]. These data show that ABCB2/B3 is functionally equivalent to a canonical ABC transporter in which NBS1 no longer hydrolyzes ATP, but suggest that ABCB2/B3 needs a nonhydrolyzing NBS1 to reach full transport activity.

Following the initial results on the role of the D‐loops, an intensive mutational analysis was performed [94]. Mutations of the D‐loop of ABCB3 did not change ATPase activity nor peptide transport compared to wild‐type. In striking contrast, mutation of the conserved aspartate residue of the D‐loop of ABCB2 resulted in an ATPase deficient transporter, while still binding MgATP and MgADP. Unexpected, and in contrast to ABCB3, the D‐loop mutant in ABCB2 converted ABCB2/B3 from a unidirectional, primary active transporter into a nucleotide‐gated facilitator that lost directionality of substrate transport. Instead substrate movements along existing concentration gradients occurred. These data emphasize the pivotal role of the D‐loop in peptide transport in ABCB2/B3 and TM287/288. However, the same mutations in the asymmetric sterol transporter ABCG5/G8 did not influence substrate transport [95]. The D‐loop is not in contact with MgATP in the closed NBD dimer, thus most likely not directly involved in the hydrolysis of ATP, which is a notion that is supported by data from ABCG5/G8. We can infer from structural, biochemical, and functional data of type I ABC exporters that the D‐loop is essential for NBD‐NBD interactions, relative NBD orientation and cooperativity between the NBSs, and thus indirectly associated with ATP hydrolysis. A reduction of the stability of the NBD dimer or a change in the preferred orientation could lead to a protein that might switch to an inward‐facing conformation after substrate binding from the extracellular site, thereby explaining the nucleotide‐gated facilitator behavior of the ABCB2 D‐loop mutant.

### ABCC1

The human ABCC subfamily is characterized by the presence of a degenerate NBS1 in all family members. ABCC1 was the first transporter of the ABCC subfamily that was shown to recognize and transport a large number of xenobiotic compounds [64, 96]. It was also demonstrated that transported substrates increase binding of ATP to the NBSs [97] and that NBS1 and NBS2 are nonequivalent [96, 97, 98]. By comparing data from radioactive labeled ATP with phosphate 32 in the α or γ position allows to discriminate between binding and hydrolysis. The γ position is cleaved off during ATP hydrolysis leaving an ADP molecule that is not radioactive, while if labeled in the α position, both ATP and ADP are radioactive. The ATP analog carrying an azido (N_3_) moiety at position 8 was shown to be functionally similar to ATP, while photo‐activation leads to chemical cross‐linking with the transporter. Combining radioactive labeling with the introduction of the azido group allows a quantification of nucleotide binding to the transporter and to determine whether hydrolysis occurred. By comparing results using 8‐azido‐α‐ATP and 8‐azido‐γ‐ATP, it was observed that ATP binds preferentially to NBS1, ATP is hydrolyzed in NBS2 [22] [99], vanadate trapping occurs mainly in NBS2 [97], and ADP increases ATP hydrolysis by ABCC1 at low concentrations, while high concentrations of ADP are inhibitory [100]. ADP is binding to both NBSs but has higher affinity for NBS2 [101], which is further enhanced by ATP. Not all ATP analogs are equivalent: While binding of a nucleotide (ATP, ADP or ATP‐γS) leads to vanadate trapping in NBS2, the frequently used ATP analog AMP‐PNP has a higher affinity for NBS1 but not able to promote vanadate trapping [102]. Importantly, vanadate induces strong labeling of 8‐azido‐α‐ATP, consistent with the posthydrolytic state, but vanadate also stimulated binding of 8‐azido‐α‐ATP, thus the prehydrolytic state [99]. Vanadate trapping of 8‐azido‐ADP in NBS2 allosterically stimulates binding of 8‐azido‐γ‐ATP to the other NBS1 [99]. Labeling by 8‐azido‐α‐ATP is much stronger than labeling with 8‐azido‐γ‐ATP [99], showing that two nucleotides are bound to ABCC1 (only one hydrolyzed), which differs from data obtained for ABCB1 [78]. NBS1 and NBS2 are allosterically coupled, and consistently, addition of nonradioactive ADP increased binding of 8‐azido‐γ‐ATP fivefold, while not affecting 8‐azido‐α‐ATP. This shows that ATP bound to the degenerate site is trapped by ADP bound to NBS2 [99].

Introduction of the canonical glutamate in place of the aspartate of the degenerate Walker B motif in NBS1 enhanced its hydrolytic capacity and the affinity for ADP, but markedly decreases transport activity, while a leucine, glutamine or asparagine mutant had little effect on function [103, 104]. Mutation of either Q‐loop glutamines to asparagines similarly reduced MgATP binding, increased *K*_M_ and *V*_max_, suggesting that the role of the Q‐loop in ABCC1 is not directly linked to ATP hydrolysis or transporter asymmetry [105]. Mutation of the H‐loop histidine in NBS1 has little effect on substrate transport by ABCC1, while mutation of the corresponding residue in NBS2 abolished transport almost completely, except for the glutamine and asparagine mutants, which maintained wild‐type like transport activity [106].

These biochemical data clearly show that under physiological conditions, ATP remains bound to NBS1 and maintains association of the NBDs, while the allosterically coupled NBS2 is hydrolyzing ATP in consecutive transport cycles. The affinity of most nucleotides is higher for NBS1. While very speculative and awaiting verification by independent experimental approaches, it is tempting to deduce from the vanadate data a backward reaction in which ABCC1 can be trapped by MgADP and vanadate without ATP hydrolysis. This would imply that NBS2 should not separate much during the transport cycle, but contemporaneously it does not mean that ABCC1 could function as an ATP synthetase.

### ABCC7

While activation by phosphorylation is a prerequisite for channel function, the focus of this review is on the role of the NBDs on ATP binding‐dependent channel opening. It has been shown that NBS2 hydrolyzes ATP, which leads to channel closure. Similar to ABCB2, isolated NBD1 forms homodimers in an ATP‐dependent manner [107]. ABC proteins including ABCC7 hydrolyze ATP on the millisecond timescale, but interestingly, ATP remains bound to the degenerate NBS1 in the full‐length ABCC7 for several minutes without being hydrolyzed or exchanged [21, 23, 24]. Kinetic analysis derived from channel recording of ABCC7 indicated that 20–30 ATPs are hydrolyzed in NBS2 before NBS1 opens [108]. An important residue for binding ATP is the tyrosine residue of the A‐loop. After mutating this A‐loop tyrosine in NBS1, the residence time of ATP became short, directly confirming that the slow kinetics is associated with ATP binding to NBS1. These data indicate that channel closing is solely controlled by NBS2 through the hydrolysis of ATP and show that NBS1 does not open during the gating cycle, thus serving as a glue that maintains close proximity between NBD1 and NBD2 at NBS1. This tight interaction and the resulting tight coupling between pore opening and formation of the tight head‐to‐tail dimer of the NBDs is mainly driven by ATP binding, which serves as an interaction hub [44]. In addition to interactions with the nucleotide, an important NBD1‐NBD2 interaction was traced to a hydrogen bond between two amino acid residues of the two opposing NBDs, R555 and T1246. These two residues of NBS2 form a hydrogen bond in the NBD dimer (open pore), but not if NBS2 dissociates (closed pore) [109, 110, 111]. This points toward a hierarchy in communication to form the tight NBD dimer required for channel gating. The tight NBD dimer dissociates after ATP hydrolysis in NBS2 and opening of NBS2, inducing the switch to the inward‐facing, closed channel conformation. Only after formation of closed NBS2, the pore re‐opens. The return to the closed NBD dimer after nucleotide exchange in NBS2 is supported and eased by the constant contact in NBS1. Therefore, NBS1 and NBS2 have diverged in function, with NBS2 having the role of changing protein geometry through ATP binding and hydrolysis, while NBS1 has a structural role by maintaining an ATP‐dependent stable NBS1 geometry, potentially serving as ATP sensor. A recent review summarizes in depth structure, gating, and regulation of ABCC7 on the molecular level and includes a summary of the underlying thermodynamics that govern channel function [112].

### TM287/288

The transported substrate of TM287/288 from the thermophilic bacterium *Thermotoga maritimus* is unknown. Under conditions of transporter overexpression, a resistance toward daunomycin was detected as well as transport of 2,7‐bis(carboxyethyl)‐5(6)‐carboxyfluorescein, while classic multidrug resistance transporter substrates such as Hoechst 33342 stimulated the ATPase activity [13]. Chemical cross‐linking of 8‐azido‐ATP showed a preference for binding to NBS1 [13], suggesting a higher affinity for MgATP in the degenerate site. Affinity measurement determined a 25‐fold difference in affinity for MgATP between NBS1 and NBS2. In contrast, the isolated NBDs of TM288 bound MgATP, while the isolated NBD of TM287 did not interact with MgATP [113].

### TmrAB

TmrAB of the thermophilic Gram‐negative eubacterium *Thermus thermophilus* has one degenerate NBS with TmrA carrying the canonical Walker B. Mutation of the canonical Walker B glutamate of TmrA to glutamine inactivates the transporter, but the transporter retains residual ATPase and transport activity [114], with a half life of the nucleotide occluded state of 24 min at 20 °C and an ATP turnover of 29 ± 1 min at room temperature. The slow rate of the mutant transporter allowed to show that substrate is transported into liposomes before ATP is hydrolyzed, therefore showing that nucleotide binding alone is sufficient for substrate translocation in TmrAB. As for most other ABC transporters with a degenerate site, insertion of a canonical glutamate into the degenerate Walker B of TmrB does not rescue an inactivated Walker B TmrA variant. ABCB11 is an exception to this rule, as insertion of a glutamate into the Walker B of the degenerate NBS1 was able to rescue the mutation of the Walker B in the canonical NBS2 [43].

### Pdr5 from *Saccharomyces cerevisiae*

Pdr5 from *S. cerevisiae* is the prototype of the pleiotropic drug resistance (PDR) subfamily of transporters [115] that are characterized by a degenerate NBS1. These ABC transporters are present only in fungi, plan, slime molds, oomycetes, and brown algae. PDRs are full‐length transporters that are characterized by an inverse domain topology in which the NBDs precede the TMDs (NBD1‐TMD1‐NBD2‐TMD2), a domain arrangement that is shared with the human ABCG subfamily. Pdr5 shows one of the largest degree of deviations from the consensus sequence, as not only the catalytically active glutamate of the Walker B motif of NBD1 and the signature sequence of NBD2 differ. Rather, all motifs of NBS1 harbor changes of functionally relevant amino acids: The lysine of the Walker A motif is replaced by a cysteine residue, the glutamate of the Walker B motif by an asparagine, and the histidine of the H‐loop by a tyrosine residue. In NBD2, the canonical sequence (LSGGQ/R) of the signature sequence or C‐loop is LNVEQ. Similar to ABCB2/B3 and ABCC1, mutations of single residues of NBS1 did not affect ATPase activity and/or transport function [116]. The same was true for substitutions of one or two sequence motifs. However, starting with replacements in three motifs, effects on ATPase activity and R6G transport were observed [117]. When all four sequence motifs (Walker A and B motifs, signature sequence, and H‐loop) of the degenerate NBS1 were replaced by canonical sequences [117], ATPase activity and R6G transport were abrogated to background levels [117], a result that is more extreme, but in principle shared with ABCB11, ABCC1, and ABCC7. This clearly demonstrates that the degenerate NBS1 has a profound influence on the function of the canonical NBS2. If results from other degenerate ABC transporters can be transferred to Pdr5, it implies that a closed NBS1 is required for ATP hydrolysis in NBS2 and to induce TMD movements. It is also tempting to speculate that movements of the TMDs are not as ‘simple’ as suggested by ‘the two site access’ model [118]. However, to address these questions in detail an *in vitro* system and structural information is required. A step toward establishing an *in vitro* system for Pdr5 has only recently been accomplished [119], but we still await structural information.

These biochemical data show clear differences in the function of NBS1 and NBS2, while the affinity for MgATP to the degenerate NBS1 is comparable to the canonical NBS2. The data for ABCC1 and ABCC7 suggest that MgATP remains bound to NBS1 over many ATP hydrolysis cycles in NBS2. Also, homodimerisation of isolated NBSs of ABCB2 in the presence of MgATP highlights the role of the degenerate NBS1 for MgATP‐dependent dimerization. Interestingly, insertion of canonical residues in the degenerate motifs of NBS1 leads to a loss of function, which contrasts with the assumption that NBS1 and NBS2 share their function. The degenerate NBS1 seems to have developed a new function that is associated with stable binding of ATP, whereby MgATP serves as a stationary hub connecting NBD1 with NBD2. It remains to be seen if NBS1 acquired the function of an ATP sensor, which regulates ATP hydrolysis in NBS2 according to cytosolic ATP levels.

## Simulations of asymmetric transporters

Simulations using isolated NBDs investigated the stabilization by nucleotides of the NBD dimer of ABCB1 [44, 45] and found that MgATP bound to both NBSs has a strong stabilizing effect (~ 42 kJ·mol^−1^), which served as interaction hub for the closely engaged NBD dimer by interacting with both NBDs. Also, MgADP stabilized the closely engaged NBD dimer, though to a lesser extent than MgATP. In contrast, the ATP hydrolysis products MgADP and Pi represented a high energy state, which primed the NBD dimer for separating NBD1 and NBD2. Simulations of the NBDs of MJ0796 showed stable NBD dimers in the presence of two ATPs, but when one of the ATP was replaced by ADP, a rotation of the helical domain relative to the core domain led to the opening of the respective NBS [120, 121].

Simulations of full‐length ABC transporters from the ABCB subfamily reported that the closed NBD dimer is only stable if ATP is present in NBS1 and NBS2 (Sav1866, MsbA, ABCB1). It was observed for ABCB1 that the NBD dimer became asymmetric in the presence of MgATP [122]. A similar asymmetry was reported for Sav1688 in some simulations [123], while others reported a symmetric NBD configuration [124, 125]. Simulations starting from the inward‐facing MsbA and ABCB1 structures were very dynamic and drifted away from the starting structure following a naturally existing energy gradient, suggesting that the wide‐open conformations of the respective starting structure were not compatible with a membrane environment [126, 127]. Asymmetry was observed in several simulations using an asymmetric nucleotide binding state. In Sav1866, the presence of ATP stabilized the closely associated NBDs, while ADP or an empty NBS led to an opening of the respective NBS [128, 129].

Extensive simulations of ATP‐bound TM287/288 observed a spontaneous conversion from the inward‐facing state to the outward‐facing state in a subset of simulations. Starting from the asymmetric inward‐facing state, he NBDs reached a configuration with symmetrically bound ATPs to the NBD dimer interface [130]. A similar structural conversion of the NBDs could be observed when associated with substrate transport: verapamil or daunorubicin, which were initially docked in the substrate‐binding pocket, were released into the extracellular leaflet of the membrane after conversion to the outward‐facing state [131].

Simulations of inward‐facing ABCC7 showed that the structures determined by cryo–EM were not stable when inserted in a membrane environment [132, 133] leading to a transition toward a more closed conformation, consistent with results from other type I exporter [126, 134]. Interestingly, simulations of ABCC7 starting from a homology model based on the inward‐facing conformation of TM287/288 [132], which harbors asymmetric NBD‐NBD contacts, resulted in an increased separation of the NBDs in the absence of ATP. These results of ABCC7 simulations (all carried out in the absence of the regulatory domain) are consistent with available data, which imply that in the absence of nucleotides, the NBDs are highly flexible and therefore should not intimately associate, but also show that the wide‐open separation of inward‐facing ABC transporter structures are too extreme for a membrane environment.

Sequence analysis, structure determination, and simulation data have shown that the TMDs of ABCC7 are asymmetric with respect to the exposed charged residues proposed to be involved in chloride conductance. A chloride conductance path was mapped from the intracellular side of the membrane to the extracellular side [68, 135]. While simulations could not directly show ion conductance, the channel was found to become filled with chloride ions, most likely because of the overall positive electrostatic potential, while only rarely a complete conductance channel formed by an increased separation between TMH6 and TMH8 [133, 135]. The simulations therefore been too short to show a complete transition to a state that is competent for ion conductance. The timescale of ATP hydrolysis in NBS1 and channel closure by reaching the inward‐facing state is even slower and thus out of reach for equilibrium molecular dynamics simulations. The use of enhanced sampling approaches, guided by available structural and biochemical information, will be needed to investigate gating movements of ABCC7.

## Transport cycle

The similarity of NBD sequences across all ABC proteins and the appearance of a large number of NBDs which were all very similar to each other stimulated the suggestion of a generalized transport cycle mechanism applicable to all ABC transporters [136, 137, 138, 139], which in light of the current structural data seems unlikely. Such a common mechanism is based on the general alternating access model proposed by Jardetzky in 1966 [118] and proposes a change between the outward‐facing and the inward‐facing state that is energized by ATP binding and hydrolysis. The switch model [85] suggests that this conformational change is associated with the switch from a high affinity state for substrates to a low affinity state and proposes that the switch is induced by ATP binding, while ATP hydrolysis is not required, because also binding of MgADP in presence of vanadate and MgAMP‐PNP leads to the formation of the low affinity state for substrate(s) [140, 141]. This does not necessarily mean that an inversion of a transport cycle is possible, i.e. synthesis of ATP. Not all transporters are equal, as the ATP analog MgAMP‐PNP induced a changed in substrate affinity in ABCB1 [142], but could not induce a transition to the outward‐facing state in TM287/288 [49]. A conformational change that is associated with nucleotide binding has now been observed in several ABC transporters [49, 94, 103, 111, 114, 140]. The processive clamp model expands on ATP binding and hydrolysis and proposes a sequential hydrolysis of two ATPs prior to NBD dissociation and before NBD reopening, which leads to a return to the inward‐facing state [32]. While suggesting sequential hydrolysis of ATP, the model proposes symmetric motions of the transporter. The occlusion‐induced switch model also relies on the switch of the TMD switching between the inward‐facing and the outward‐facing state [118] and proposes the hydrolysis of two ATPs per transport cycle: the first ATP is hydrolyzed for the transition from the high affinity to the low affinity state, while the second ATP hydrolysis is required for resetting the transporter to the ground state.

In contrast to the above models that propose a full separation of the NBDs during the transport cycle, the sequential model [143] requires that the NBDs remain in contact, one ATP is hydrolyzed per transport cycle, suggesting strict alternation between NBS1 and NBS2 and associated conformational changes. Similarly, the constant contact model proposes that the NBDs remain in contact throughout the transport cycle [121] and suggests that one ATP is hydrolyzed per transported substrate as well as an inherent asymmetry throughout the transport cycle, but does not require strict alternation between NBS1 and NBS2.

Most of these models were proposed before full transporter structures became available in multiple conformations and were largely derived from ABC transporters with two canonical NBSs. These models are generally not fully compatible with all the data derived from transporters harboring a degenerate NBS1, suggesting a variation of the transport cycle with a noncanonical NBS. Some transport models were also specifically proposed for transporters harboring a degenerate site. A simplistic transport cycle model was proposed for ABCC1 using information from several cryo‐EM structures [66] and predicts substrate binding to the wide‐open inward‐facing transporter, followed by ATP binding and NBD association and a switch to the outward‐open state. In the third step, the transport cycle concludes by ATP hydrolysis and ADP release. This model neglects asymmetry, but is also incompatible with biochemical data from ABCC1 which show that full separation of NBS1 can be excluded [96, 97, 98].

In the transport model proposed for heterodimeric TM287/288 [13], ATP remains bound to NBS1 throughout the entire transport cycle. Binding of substrate triggers binding of ATP to NBS2 and an accompanying switch to the outward‐facing state, in which substrate is released. Subsequent hydrolysis of ATP in NBS2 resets the system to the inward‐facing conformation. Only occasionally, however, ATP in NBS1 becomes hydrolyzed and ADP dissociates after opening of NBS1. The model is supported by the crystal structure of the apo form [48] that shows partially engaged NBDs. Binding of ATP to NBS1 did not re‐arrange the conformation of the TMDs as demonstrated by EPR measurements. A key element of this behavior was assigned to the D‐loop motif of the degenerate NBS, which links together both NBDs. Interestingly, a mutation of the conserved aspartate to alanine was tolerated in the degenerate, but not in the canonical NBS. These results point to an allosteric regulation of the consensus NBS by the degenerate NBS and indicate that the degenerate NBS prevents the NBDs to completely disengage.

The transport model proposed for heterodimeric TmrAB deviates from the constant contact model as proposed for TM287/288. To derive the model, eight structures of TmrAB reconstituted into nanodiscs were used to trace a continuous path through the transport cycle [52]. The model of the transport cycle requires substrate binding to the inward‐open conformation of pseudoseparated NBDs as they remaining in contact by an unusual extended helix at the C terminus of the NBDs. ADP is exchanged for ATP in this state, while ATP is proposed to continuously bind/unbind until the closed NBD dimer forms. This model is therefore not compatible with the data derived for ABCC1 and ABCC7. NBD dimerization triggers a switch to the occluded and subsequently the outward‐facing state. Interestingly, this conformational shift can also occur in the absence of substrate. The occluded state and the outward‐facing state are proposed to both be capable of hydrolyzing ATP in NBS2. These two conformations are in equilibrium before and after substrate release and are suggested to keep oscillating until P_i_ is released, which triggers asymmetric opening of the NBDs to conclude the transport cycle. The rate limiting step is the exchange of the nucleotide triphosphate in the inward‐facing conformation [52] that lead to an accumulation of the inward‐facing conformation, while the affinity of nucleotides to the separated NBDs must be high, because of the slow off rate, which is unusual for ABC exporters. As nucleotide exchange is proposed for both NBSs, this model proposes a mechanism that would in theory be applicable to transporters with two active NBSs and also to transporters with a degenerate NBS1. The pseudoseparation of the two NBDs stabilized by the additional helix and their strong interactions and its unusual NBD geometry suggest that motions and conformations observed for TmrAB might not be representative for every type I exporter.

The alternating access model [136] has been proposed as a generalized model to describe transport of substrates across the membrane. The model posits that the transporter has a substrate‐binding site within the path of translocation, separated by gates toward either side of the membrane. A key feature of this model is that the substrate permeation path is never contemporaneously open to both sides of the membrane. It has now been established that secondary active transporters follow this generalized model, and their substrate‐binding site is approximately halfway through the membrane. Data are accumulating from structural and biochemical studies that primary active ABC transporters are also consistent with this generalized scheme of substrate translocation, with ABCC7 being a well‐known exception that functions as an ATP‐gated chloride ion channel by opening both gates.

We can infer from the available structures of ABC transporters that not all ABC exporters use the same mechanics for cargo translocation, which is linked to differences in transporter folds. The available structures suggest that in the ABCB and ABCC subfamilies, the main axis of rotation runs approximately parallel to the main axis of the transporter and therefore normal to the membrane, while the conformations observed for ABCG2 [144] and ABCG5/G8 [145] show a rotational axis that is flipped by 90° and runs parallel to the membrane and to the NBD‐NBD interface. This structural difference leads to very different structural changes of the NBDs following ATP hydrolysis and their propagation to the TMDs.

## Summary and Perspectives

ATP‐binding cassette transporters are characterized by a twofold symmetry axis which implies that every structural element is present twice. NBS1 and NBS2 are identical in homodimeric transporters and remain very similar in heterodimeric or full transporters. Of note, 21 of the 29 human nonhomodimeric ABC transporters have a degenerate NBS. It seems therefore likely that the degenerate NBS has an evolutionary advantage.

### Division of labor between degenerate NBS1 and NBS2

We can infer from sequence alignments that the degenerate NBS has acquired a new function, because it remained under evolutionary pressure, and its sequence has not further diverged as would be expected for a site that has lost any function. The consistent motions and concurrent direction of rotation of the NBDs relative to each other (Fig. 3) suggest that the asymmetry of the NBDs is also reflected by the mechanics of the TMDs. The relative orientation of the NBDs is asymmetric in the apo structures of type I exporters as they lack stabilization by nucleotides. All these structures show the same direction of rotation, with TmrAB being an exception. The degenerate NBS could act as ATP sensor, but this interpretation remains uncertain because also canonical NBSs could serve as ATP sensors. ATP bound to NBS1 seems to serve as hinge for motions leading to NBS2 closure and opening following ATP binding and hydrolysis. Such a stable hinge could increase transporter efficiency and/or the use of ATP. Consistently, when canonical residues were introduced in the degenerate NBSs, transporters showed no change in function nor a loss of function, but never a gain in function. Thus, the degenerate NBS1 has assumed a structural and mechanical role, while the active ATPase of NBS2 energizes the transport cycle. Evolution can thus optimize for the division of roles, while in ABC transporters with two active ATPases, both NBSs need to fulfill both roles.

### Role of TMD motions

Motions in the NBDs are tightly coupled to motions in the TMDs to allow for substrate transport. In transporters with a degenerate NBS, the asymmetry of the NBDs most likely translates into asymmetric motions of the TMDs (Fig. 3). If TMDs do not reach a state that shows a wide‐open outer gate as first observed for Sav1866 [12], but opens a gate just large enough for the transported substrate, small motions have the advantage of requiring less energy input because of smaller perturbations of the membrane environment due to smaller conformational changes of the TMDs. The presence of only a small opening controlled by a gate exists very likely in ABCB1, as residues in TMH6 and 12 become never accessible to extracellular hydrophilic probes [146, 147, 148]. Available data are insufficient to clearly show whether type I exporters have one central gate or two symmetry‐related extracellular gates. The chloride channel ABCC7 seems to have a noncentral narrow gate located between TMH6 and TMH1 [68, 149]. Mutational and biochemical data suggest that two small gates are most likely present in ABCB1, because introducing positively charged arginine residues in symmetry‐related position of TMH2 and 8, combined with nearby mutations, selectively affected the transport of charged substrates [150, 151, 152], thus implying two symmetry‐related gates. Structures of human ABCB1 [54] show a bound taxol that is large enough to fill the entire substrate‐binding cavity. In contrast, two zosuquidar molecules, well‐known inhibitors, bind decentral to one large binding cavity. In mouse ABCB1, two symmetry‐related, yet clearly separated binding sites were identified for the smaller 2,4‐dibromophenyl 2,4,6‐tribromophenyl ether (BDE‐100) [153]. Asymmetry in the TMDs and the NBDs avoids a tug of war between NBS1 and NBS2 in response to substrate binding to one of the two pseudosymmetric substrate‐binding sites in the TMDs, thus preventing futile cycles caused by occlusion of ATP in the NBS that is not dedicated to the position of the bound substrate. While the discussion on TMD asymmetry is largely speculative and awaits experimental proof, the structures of TM287/288, TmrAB and ABCC7, as well as a large body of biochemical data, support the notion of correlated asymmetry in TMDs and NBDs.

### Directionality of substrate transport

One of the key differences between primary active and secondary active transporters is the directionality of transport. In the latter, directionality is controlled by the concentration gradient of the cotransported ion and an inverted transport direction can be physiological relevant. In contrast, ATP‐driven, primary active transporters show unidirectional transport. Two types of mutations were reported, which converted unidirectional ABC transporters to a bidirectional ATP‐gated facilitator: (a) mutations in the putative substrate‐binding/translocation path of the TMD of ABCB1 resulted in a transporter variant that shows concentration gradient dependent substrate translocation [152], and (b) mutations of the D‐loop of ABCB2/B3 [94] and ABCB9 [154] converted these transporters to ATP‐gated substrate translocation facilitator.

While the results for the different ABCB1 variants could potentially be explained by increased access from the extracellular site or a lack of gate closure, thereby compromising directionality by enhanced accessibility to the substrate‐binding cavity from the extracellular site, the effect of the D‐loop mutants is more likely linked to ATP‐dependent NBD dimerization. This interpretation is consistent with the notion that nucleotides control transporter conformation such as the exporter is inward‐facing in the apo state, while outward‐facing, when two ATP are bound. Sequence comparison (Fig. 2) shows an increased conservation of the D‐loop, if both NBSs are capable of hydrolyzing ATP. The importance of the D‐loop for NBD dimerization and therefore regulation of the overall transporter geometry suggest that it might be easier to maintain transport directionality in exporters with a degenerate NBS, which more efficiently regulates nucleotide hydrolysis in response to substrate binding, or because facilitating the development of an improved external gate that prevents rebinding and reverse transport.

### Could one mechanism serve them all?

It remains debated if all ABC exporters follow the same transport cycle. As outlined above, it is unlikely that type I ABC exporters (ABCB/C/D subfamilies) share the mechanism of transport with type II ABC exporters (ABCA/G subfamilies). When focusing on type I ABC exporters and specifically on the ABCB and ABCC subfamilies, these transporters share a number of features: The difference in sequence between the ABCB and ABCC subfamilies is small and, accordingly, structure determinations have shown that they share the same fold. While all transporters from the ABCC subfamily have a degenerate NBS1, also some belonging to the ABCB subfamily harbor a degenerate NBS1. The conformations of the closed NBD dimer are basically the same, if a nonhydrolyzable ATP analog is bound or if the glutamate of the Walker B motif is mutated. In contrast to the structural similarity, biochemical data have clearly shown that wild‐type transporters harboring a degenerate NBS (such as ABCC1 [101]) bind two nucleotides under physiological conditions, whereas those with two canonical NBSs (such as ABCB1 [78] seem to bind only one nucleotide.

**Fig. 5:**
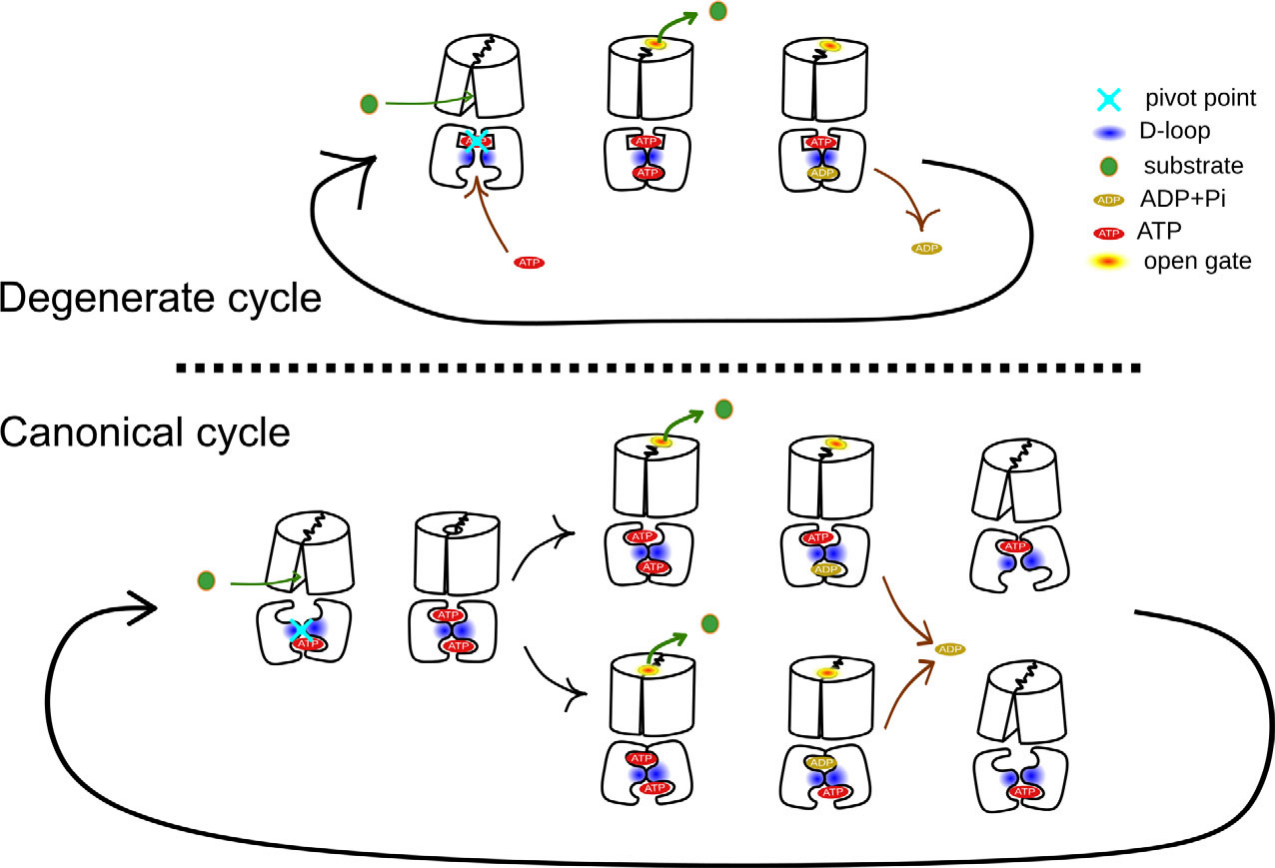
Model of the ABC exporter transport cycle. ABC transporters from the ABCB and the ABCC subfamily share the same sequence of conformational changes controlling the transport cycle and the structural asymmetry. While ABC proteins with a degenerate NBS (upper cycle) show NBD motions similar to a clamp‐shell by closing/opening only NBS2 during ATP binding, hydrolysis and ADP + Pi release, transporters with two canonical NBSs might function as a seesaw by occluding and hydrolyzing ATP in either NBS, but never simultaneously. The two types of NBDs might differ in the position of the hinge point and in their gates that open to release substrate to the extracellular site.

With respect to the human transporters, these data could be interpreted in the following way: The motions of the NBDs of transporters with a degenerate NBS operate similar to a clamshell, while transporters with two canonical NBSs follow a seesaw motion of NBD rotation, but without a clear sequential order (Fig. 5). A transport cycle starts with an asymmetric inward‐facing transporter that has one bound ATP, which remains from the previous transport cycle. This ATP resides in NBS1 in ABC exporters with a degenerate site, while it could be NBS1 or NBS2 in transporters with two canonical NBSs. Subsequently, ATP binds to the open and empty NBS, while substrate can bind to the TMDs. Subsequent motions lead to ATP occlusion and to a transition to the outward‐facing state. It was biochemically shown that ATP occlusion is asymmetric in transporters with a degenerate NBS, but also in ABC transporters with two canonical NBSs (see section ‘Biochemical evidence for asymmetry’ for further details). Basal ATPase activity is associated with ATP occlusion and transition to the outward‐facing state in the absences of substrate. Binding of substrate and of ATP contribute to the transition, cooperatively enhancing the ATPase activity in most transporters. The outer gate opens in the outward‐facing state to release substrate. While for some bacterial type I exporters widely separated wings were detected, this is unlikely for the majority of human ABC type I exporters that translocate hydrophobic substrates, including the lipid and multidrug resistance transporters. Such large motions are also not necessary, because typical substrates are small. These extensive TMD motions would be energetically very expensive requiring that the TMDs push against the surrounding membrane, while exposing larger hydrophobic surfaces to a water environment. The outward‐facing state is most likely also asymmetric in the TMDs, as apparent for ABCC7, but also biochemically shown for ABCB1. The hydrolysis of ATP leads to a high energy state [45] that promotes immediate separation of that NBS. For transporters with a degenerate NBS, this process is inherently asymmetric as only NBS2 is hydrolysis competent. In all cases, under physiological conditions, the NBDs should only show rare, if any, complete separation of the NBDs, whereby some transporters might deviate, because of extra structural features, such as TmrAB.

ATP in NBS1 is most likely serving as a hinge point for the transport cycle in transporters with a degenerate site, where it remains stably bound for 20–30 cycles. Comparing transporters with one or two canonical ATPases, a single loop through the transport cycle could be very similar, except that hydrolysis can occur stochastically in either of the two NBSs, if NBS1 is also ATP hydrolysis competent. The nonhydrolyzed ATP in the second NBS could serve as a hinge to guide the rotational motions. The fact that two nucleotides could not simultaneously be trapped in both NBSs of ABC transporters with two canonical NBSs suggests that ATP in the second NBS can probably not or less efficiently serve as a motion‐stabilizing hinge. Instead, both NBSs primarily serve the role of providing energy for ATP binding and hydrolysis, thus suggesting that full NBD separation might occur more frequently, if ADP/ATP exchange is not fast enough. It seems plausible that the D‐loop in the center of the NBD‐NBD interface could contribute to the hinge and/or serve as spacer, thereby shifting the hinge point from ATP in NBS1 of degenerate NBSs toward the center of the NBD dimer in transporters with two canonical NBSs.

In conclusion, the available structural and dynamic data suggest a common conformational path through the transport cycle, though ABC transporters with a degenerate NBS have a stronger tendency for rotation relative to each other. Biochemical data are not clear enough to affirm or reject the hypothesis of a partially shared mechanism. From the currently available data, we can infer that ATP in the degenerate NBS1 has acquired the role of stabilizing the closed NBD dimer and to serve as a hinge for conformational changes, although it remains unclear if ATP plays the same role in transporters with two canonical NBSs.

## Author contributions

TS, RG, and LS drafted the manuscript. RG generated the figures. TS and LS edited the manuscript. The final version was approved by all authors.
